# Perceptions, careseeking, and experiences pertaining to non-cephalic births in rural Sarlahi District, Nepal: a qualitative study

**DOI:** 10.1186/s12884-018-1724-2

**Published:** 2018-04-10

**Authors:** Naoko Kozuki, Luke C. Mullany, Subarna K. Khatry, James M. Tielsch, Steven C. LeClerq, Caitlin E. Kennedy, Joanne Katz

**Affiliations:** 1 Department of International Health, Johns Hopkins Bloomberg School of Public Health, Baltimore, USA; 2 Nepal Nutrition Intervention Project, Sarlahi, Nepal; 3 Department of Global Health, George Washington University School of Public Health and Health Services, Washington, DC USA

**Keywords:** LMIC, Non-cephalic presentation, Intrapartum complications, Neonatal, Fetal, Maternal, Careseeking, Care seeking

## Abstract

**Background:**

In low-resource settings, a significant proportion of fetal, neonatal, and maternal deaths can be attributed to intrapartum-related complications. Certain risk factors, such as non-cephalic presentation, have a particularly high risk of complications. This qualitative study describes experiences around non-cephalic births and highlights existing perceptions and care-seeking behavior specific to non-cephalic presentation in rural Sarlahi District, Nepal.

**Methods:**

We conducted in-depth interviews with 34 individuals, including women who recently gave birth to a non-cephalic infant and female decision-makers in their households. We also conducted two focus groups with mothers (have two or more children, with at least one child under age five) and two focus groups with grandmothers in the community.

**Results:**

Several women described scenes of obstructed labor and practices like provision of unspecified injections early in labor to assist with the delivery. There were reports of arduous care-seeking processes from primary health centers to tertiary facilities, and mixed quality of care among home birth attendants and facility-based health workers respectively. Very few women were aware of the fetal presentation prior to delivery, and we identified no consistent understanding among participants of the risks of and care strategies for non-cephalic births. Risk perception around non-cephalic presentation varied widely. Some participants were acutely aware of potential dangers, while others had not heard of non-cephalic birth. Many interviewees said that the position in which a pregnant woman sleeps could impact the fetal position. Several participants had either taken or heard of medication intended to rotate the fetus into the correct position.

**Conclusions:**

Our findings suggest the mixed quality of and access to care associated with non-cephalic birth and a lack of consistent understanding of the risk of and care for non-cephalic births in rural Nepal. The high risk of the condition and the recommended tertiary care present a dilemma in low-resource settings; the logistical difficulties and the mixed quality of care make care-seeking and referral decisions complex. While public health stakeholders strive to improve the quality of and access to the formal health system, those players must also be sensitive to the potential negative implications of promoting institutional care-seeking.

**Electronic supplementary material:**

The online version of this article (10.1186/s12884-018-1724-2) contains supplementary material, which is available to authorized users.

## Background

Globally, 2.7 million neonatal deaths [1] and 2.6 million third-trimester stillbirths [2] occur annually, a large majority in low- and middle-income countries. Of those, 1.2 million occur during labor and an additional 1 million die within the first day of life, mostly due to insults during labor and delivery [3]. A focus on the intrapartum period holds great potential to prevent not only fetal, neonatal, and maternal mortality, but also neurocognitive impairment and morbidities resulting from conditions such as neonatal encephalopathy [4]. Approximately 40% of women worldwide still deliver at home and a majority of these births do not have a skilled birth attendant present, making labor and delivery all the more risky [5]. It may be valuable to identify and refer in the antepartum period women at particularly high risk of complications.

For instance, fetuses in non-cephalic presentation (presenting with a body part other than its head first, e.g. breech) have heightened risk of intrapartum-related complications and subsequent fetal and neonatal death [3, 6–8]. Specifically in the same context in rural Nepal as this paper reports on, non-cephalic births had 13-fold increased risk (adjusted risk ratio 12.52, 95% CI: 7.86–19.95) compared to cephalic births, and a nearly five-fold increased risk (adjusted risk ratio, 4.57, 95% CI: 1.44–14.50) of early neonatal mortality.

Existing epidemiologic data associate high mortality and morbidity with non-cephalic birth, but there are limited data available on perceptions and care-seeking associated with non-cephalic presentation in low-resource settings. One ethnographic study conducted in the 1980s on Nepal’s Magar ethnic group described the perceived association between breech births and adverse birth outcomes. If a pregnant woman was suspected to have a breech fetus, attempts would be made to rotate the fetus, and if unsuccessful, it was said that the fetus bites the mother’s heart, resulting in maternal and fetal death [9]. Another study from Jamaica mentioned how several mothers and their social networks expressed acute fear of the dangers associated with breech deliveries, and also reported that a few women only arrived at the hospital in advanced labor despite being aware of malpresentation earlier in pregnancy [10].

To better understand care-seeking patterns and barriers to care for non-cephalic presentation, we describe the intrapartum conditions of women who recently gave non-cephalic birth in rural Sarlahi District, Nepal, and highlight existing perceptions and care-seeking behavior specific to non-cephalic presentation. Through interviews with women who gave non-cephalic birth in the past year, interviews with female decision-makers in their households, and focus groups with women in the community, we seek to identify the barriers to care-seeking among pregnant women giving non-cephalic birth.

## Methods

This study was conducted in rural Sarlahi District, Nepal, from November 2014 to January 2015 [11]. The study was nested in a cluster randomized community-based trial examining how neonatal massage using sunflower seed oil, compared to traditionally-used mustard seed oil, impacts neonatal mortality and morbidity (ClinicalTrials.gov NCT01177111). We do not believe the intervention of this parent trial, which was provided after delivery, affected our findings here.

As part of routine follow-up of recently-delivered women enrolled in the trial, they were asked about fetal presentation (which part of the fetal body presented first during delivery). For this qualitative study, we used those responses to identify women who gave non-cephalic birth between two and twelve months prior to the time of interview. An interview guide was created with questions organized into the following topic areas: background characteristics of the interviewee, conditions during pregnancy, conditions during labor and delivery, and non-cephalic presentation. The interview guide was created in English, then translated into Nepali with input from the local staff, then translated into Maithili, the local language in the study area. The Maithili guide was also verbally back-translated into English to check for errors.

To obtain the individual perspectives of the women who gave birth (referred to as the “woman” hereafter) and the female decision-makers of their households (referred to hereafter by her relation to the woman, e.g. “mother” or “mother-in-law”), we conducted independent, but simultaneous in-depth interviews (IDI) with these two individuals in one household. Local female staff trained in qualitative data collection first conducted eight pairs of interviews. The woman and the decision-maker were interviewed at their homes in separate rooms. The first author, who is not Nepali, was present at the home but did not sit in on the interviews to ensure that an outsider's presence not create discomfort or distraction for the interviewees. Each interview was audio-recorded.

Following each pair of interviews, the first author conducted debriefings with the interviewers to summarize content, highlight common or discordant themes between the respondents, revise questions for comprehension, and discuss any difficulties. In addition to these debriefings, more extensive quality assurance activities were conducted for the earlier interviews; randomly selected portions of the first four paired audio recordings were reviewed as a group for any concerns related to phrasing of questions, tone of voice, and improper or lack of probing. Also, the interviews were temporarily halted after the first two pairs to await the full English translation of the transcripts. The first author identified relevant issues in the full translations and debriefed with the interviewers before proceeding with subsequent interviews.

After eight paired interviews, we determined that conducting two interviews per household did not provide significant additional insight, and that the decision-maker often provided more relevant information. Thus, subsequent interviews were conducted solely with the primary female decision-maker if available, and if not, with the woman herself. We continued debriefings following each interview and conducted interviews until saturation was reached, defined as no further additions to the codebook, for a final total of 34 interviews from 26 households. Male decision-makers were not interviewed due to concerns of cultural appropriateness of our qualitative research staff (married women in their twenties) interviewing male adults.

In order to better understand how the general community perceives the issue of non-cephalic presentation, we also conducted two focus group discussions (FGD) with mothers (inclusion criterion: have two or more children, with at least one under five years of age) and with grandmothers in the community (inclusion criterion: have at least one grandchild) respectively. The criteria of two or more children was set to allow for women to contribute information from several pregnancies, especially if a woman experienced both cephalic and non-cephalic births. One facilitator and one notetaker partook in each FGD. These audio-recorded discussions focused on general pregnancy care, fetal presentation, and preferences for delivery location. Debriefings between the first author and the data collectors were also conducted after each FGD.

We received informed verbal consent from all individuals participating in the IDIs and the FGDs. The IDI and FGD guides (in English and in the local language of Maithili) are available in Additional files 1 and 2 respectively.

Our study area consists predominantly of one ethnic and religious group. All eligible women belonged to the Madheshi ethnic group (a group that originated in north India and migrated into the southern plains of Nepal), and of the 26 families interviewed, 25 were Hindu and one was Muslim. IDIs and FGDs were conducted in Maithili, the language of the Madheshis. The interviewers/facilitators were locally resident Madheshi women with high school education, and spoke Maithili and Nepali fluently. The interviewers received a one-month training on qualitative data collection from Transcultural Psychosocial Organization Nepal, an NGO that supports psychosocial and mental well-being of vulnerable subpopulations.

IDI and FGD recordings were first transcribed from Maithili to Nepali by the interviewers/facilitators themselves. The transcripts were sent to Nepali translators based in Kathmandu for translation from Nepali to English. For the first four interviews, the translations were checked page by page for accuracy against the Nepali transcripts. For all other translations, the first author met with the qualitative research coordinator after one read-through of the translations for clarification. Recordings, transcripts, and translations were all de-identified, and labeled with an interview number.

The transcripts underwent an iterative coding process using Atlas.ti. The codebook started with thematic codes reflective of the major themes in the interview guides, then emergent codes were added. All transcripts were then coded and reviewed a second time. To organize the coded data, a matrix was created with major themes in rows and the individual interviews in columns. The codebook development and the coding were all conducted by the primary author. Findings arising from each interview were summarized in the matrix, and representative quotes were extracted. Findings were compared across the interviews for common or divergent perspectives under each theme. For main conclusions drawn from the interviews, disconfirming cases were sought for quality assurance, and the findings were appropriately revised based on that process.

## Results

We first interviewed eight women and the female decision-makers from their households: six mothers-in-law, one biological sister who was also a sister-in-law by marriage, and a grandmother-in-law (the woman’s husband’s grandmother). The subsequent one-per-household interviews consisted of six women, six mothers, five mothers-in-law, and one sister-in-law. The women who gave birth ranged from age 16 to 35 at the time of interview (median 23 years) and the number of previous pregnancies ranged from 0 to 5 (median 2). Age at first marriage ranged from 13 to 18 years (median 16 years). Only four women had any education, of whom only one had completed high school. For the four focus groups, both the mother focus groups had eight attendees, and the grandmother focus groups had eight and seven attendees respectively.

### Conditions during labor and delivery related to non-cephalic presentation

Most families described the fetus getting “stuck” during labor and delivery. Many mentioned the fetus hanging by the neck, with the body or the head getting stuck after its lower extremities presented. One woman compared the condition to hanging by a noose. Another woman said:

The whole of the heel of one foot came out while we were going to [government health facility]. The baby’s foot stretched inside like our foot would stretch if our foot had slipped inside a pothole in the road. It would stretch if we tried to take it out with our hands. It would not come down. I could feel it all.

The same woman added that months after delivery, she still experienced so much pain that it felt like the fetus was still stuck. Her grandmother-in-law compared the situation with what she would expect in a normal cephalic delivery, stating that “if the baby were normal, it would just slip and drop down.”

Many households described receiving injections from birth attendants, both at home and at a facility, to induce labor following such obstruction. The contents of the injections were never identified, but several women said that they provided strength and energy for delivery. One mother-in-law indicated their purpose as widening the vaginal opening. One woman reported receiving four injections just in the intrapartum period to address the obstruction. Focus groups indicated that receipt of injections was common across many women’s deliveries, regardless of fetal presentation. In both IDIs and FGDs, women stated that injections are given after labor pain begins, which differs from the clinically recommended uterotonic injections given during the third stage of labor to prevent postpartum hemorrhage. Episiotomies, known locally as a “small operation” to distinguish it from the “big operation” of a Cesarean section, were also common.

Just over half of the interviewees reported home deliveries. In multiple cases, birth attendants/family members pressed on the woman’s stomach to aid delivery, and in other cases, birth attendants and family members stuck their hands into the vagina to pull the fetal head out. Another woman described her aunt pressing on her stomach when the contractions stopped after half of the fetal body was delivered. One mother reported that she pulled the fetal leg in a way that significantly increased her daughter’s pain. It is unclear to what extent these practices were clinically sound or harmful.

One woman reported a negative experience at a health facility, while others did not comment on the quality of care. One woman and her mother-in-law eagerly and angrily described conditions during delivery at a tertiary health care facility, with the hope that our research staff could play a role in addressing these issues. The woman described the nurses as having “pulled my baby like pulling old stuff from a sack.” A doctor who arrived later reprimanded the nurses, and following delivery, referred the infant to Kathmandu, where he/she subsequently died. The same woman was also only referred from the primary facility to the tertiary facility for non-cephalic presentation in the morning after being admitted the previous evening. The mother-in-law angrily reported that if they had been told in the evening about the condition, they would have sought higher-level care sooner.

Several individuals reported an arduous care-seeking process either for the mother during labor or for the neonate after birth, being taken or referred from one facility to another in an area with poor roads and access to transportation. Several lower-level government facilities in the study area have a policy of immediately referring non-cephalic cases to tertiary facilities, although the protocol is not standardized [12]. In a handful of situations, families asked staff at the lower-level facility to try handling the birth. In a few situations, the families were asked to sign what appeared to be a liability form and permission from the guardian before the facility proceeded to care for the woman. Another woman who was referred to a tertiary facility complained, “They just said that they couldn’t do the delivery in the facility without giving any concrete reasons.” One referred family stopped in the district capital about an hour’s drive away to receive an ultrasound exam to confirm the fetal position, then continued on to a facility in India that was an additional hour away. A few families sought care for their infants in one or more local facilities before proceeding to Kathmandu (a minimum six-hour drive, often longer) for higher-level care.

### Antepartum diagnosis

Only two women knew through an antepartum ultrasound exam that their fetus was in a non-cephalic position. A third woman received an ultrasound exam in the eighth month for the purposes of fetal sex determination. Her mother-in-law was aware that fetal position could be detected through ultrasonography, but indicated that the doctor did not tell them anything about fetal presentation during their ultrasound exam. Only a few women acknowledged ultrasonography as a method of identifying the fetal position. A few interviewees were puzzled by questions regarding antepartum diagnosis of fetal presentation, as they did not understand how they could have detected the position when the fetus was still inside the womb and thus not visible. Some women suspected non-cephalic position from the physical feel during pregnancy, and several women were diagnosed inaccurately or possibly diagnosed too early in pregnancy. One woman said a traditional birth attendant falsely told her that she was pregnant with twins, and others described traditional birth attendants mistaking the fetal buttocks for the head right around the start of labor or not noticing the non-cephalic presentation until well into labor. One woman said, “We don’t know how the baby got to be in the incorrect, upside-down position,” as a village midwife (*hatkini*) had put her hand into the vagina at the beginning of labor and had declared that the fetus was in correct position. One woman had been told during an antenatal check-up with a village “doctor” (not a certified doctor) that she had a non-cephalic fetus, but later was told the contrary at a health facility. Based on that information, her family did not take her to a health facility at the time of delivery. In FGDs, ultrasonography and a physical exam by health personnel were mentioned as possible ways of diagnosing non-cephalic presentation, although the latter was not emphasized in the mothers’ focus groups. In one of the grandmothers’ focus groups, many participants expressed awareness of ultrasonography as a tool for diagnosing fetal position, but they emphasized the associated expenses over the perceived clinical benefits. Very few appeared to know the benefits of ultrasonography beyond determining fetal position and sex.

### Risk perception

We did not identify consistent perceptions held by our interviewees toward non-cephalic presentation and its associated health risks. Some participants knew that non-cephalic deliveries were dangerous, and the acuteness of that risk perception ranged across individuals. A mother-in-law related, “[The woman’s] confidence broke down when she heard that the baby was upside down. She would not have become so nervous if the baby was normal.” Another mother-in-law also relayed a similar sense of concern and panic when the fetus presented feet first, stating that she prayed and promised offerings to gods and goddesses for the health and survival of the baby, or if not, at least for the health and survival of her daughter-in-law. In both IDIs and FGDs, some women expressed concern about the possibility of a non-cephalic fetus getting stuck, and that the mother and/or child could die in the process. A majority of women in a grandmothers’ focus group stressed how dangerous non-cephalic births are and also how unpredictable they are in terms of survival of the mother or the child. Yet they all agreed that the delivery could be done at home, until a complication occurred. These concerns were not unanimously held. Some focus group participants stated that they did not know how to answer some of the posed questions when they had never experienced a non-cephalic birth before, and participants in one focus group often deferred to one woman who had a previous non-cephalic birth.

Some interviewees had never heard anything pertaining to non-cephalic presentation prior to their experience during the delivery in question. One mother-in-law indicated, “Till this day, I hadn’t heard of an upside down baby, nor had I seen or heard anything about it. I don’t know how it happened.” She subsequently noted that she would have taken her daughter-in-law to a facility had she known that the fetus was upside down. One woman indicated that the family was simply taken by surprise: “When we saw that the legs of the baby were coming out first, we were kind of shocked and could not think of how the baby was going to be born.” In another scenario, a sister-in-law reported confusion about what should be done; a traditional health worker indicated that a breech fetus could be delivered at home, while neighbors said the opposite and implored the family to take the woman to a facility. It appeared that in many cases, it was not until birth attendants or family members sensed that the labor was prolonged that they chose to seek care outside the home, and not necessarily at the initial point when they recognized that the fetus was breech. Even among those who had minimal exposure to the concept of non-cephalic presentation, there was a pervasive theme that they would have sought care if they had known about the condition before delivery.

Non-cephalic presentation did not appear to be a systematic part of risk communication during antenatal or intrapartum care. One woman noted, “I know the pain of losing a child; however I never knew about breech delivery. If I had known that my baby was not in the normal position I would have done something. The doctors didn’t even tell me once or gave any hints about it.” A few women even showed a lack of knowledge on which way a fetus is supposed to present in a normal scenario; one noted that she did not know that a baby is supposed to present head first until she delivered her first baby. That said, even those who did receive the risk communication did not necessarily seek intrapartum care; following an ultrasound diagnosis of a fetus in transverse lie (fetus lying horizontally), a doctor instructed one mother to arrive ten days before the delivery date to be admitted, but she insisted on a home delivery and continued to do so during the intrapartum period, despite family members’ insistence on heading to a facility.

Risk perception was strongest among families who had a previous non-cephalic delivery and had a related complication. Those with previous negative experiences sought care and had a pervasive sense of fear in their rhetoric and behavior. One spent a significant amount of money on a traditional healer to “prevent the umbilical cord from coming out first again,” while others sought facility care immediately upon discovering at the start of labor that the fetus was in non-cephalic position. Similarly, participants who had heard of negative consequences of non-cephalic birth from neighbors or from other sources generally sought care, and this was described in the FGDs as well. One woman said, “Everyone in my family got scared seeing the breech delivery. One of the people in our village also had a breech delivery and her child died. My family members were very concerned so they kept calling the doctors.” Those who had a complicated home delivery indicated that they would deliver at a facility the subsequent time. In contrast, a few women in both IDIs and FGDs who had a previous non-cephalic delivery but had no complications did not convey a strong sense of concern regarding non-cephalic deliveries.

### Cause of and treatment for non-cephalic presentation

The most common cause of non-cephalic presentation described by participants in both the IDIs and the FGDs was a pregnant woman’s sleeping position. Several individuals indicated that rolling over while lying down, without getting up first to turn over, causes the fetus to turn. A few others indicated that sleeping on one’s back or on the side will cause the fetus to turn upside down. Several indicated that they heard this from “doctors,” though it was unclear what level health worker they were speaking of. While this cause was often mentioned, many respondents needed probing before bringing up this information, suggesting that the topic of either non-cephalic presentation and/or its cause was not particularly salient. Some seemed to indicate only that they had heard of this before, but not necessarily that they believed it. Furthermore, several participants in the mothers’ focus groups indicated that these beliefs were just held by older generations. Other participants indicated that the position of a fetus is simply god’s will, and this theme of “mercy of god” and “fate” recurred in the grandmothers’ focus groups.

Many individuals described medication that moves the fetus into proper position. One woman reported taking up to seven medications. The mothers’ focus groups also described such medication, but qualified it by claiming that there is no guarantee that the fetus will rotate. In the grandmothers’ focus groups, some participants raised the ability of local health attendants to both detect and to rotate a non-cephalic fetus in the womb, while the role of a local health attendant was not emphasized in the mothers’ focus groups.

The concept of pregnancy care was also fluid, in that there was minimal linguistic differentiation made among formal or informal health care in the community. While there are specific traditional roles, like traditional healers (*dhami jankri*) or traditional birth attendants (*hatkini)*, who had distinct names and responsibilities and were clearly distinguishable by the interviewees, the interviewees also very freely used the English word “doctor” to identify health care workers, encompassing certified MBBS doctors, certified health workers who are not MBBS doctors, informal health workers found at the local marketplace, and also our own non-clinically trained study staff. Across several interviews, interviewees asked our staff questions regarding non-cephalic presentation, assuming they would know more as “doctors.”

## Discussion

Non-cephalic presentation has an incidence of 3–4% at term [13], making it a relatively rare condition compared to other pregnancy-related health issues such as anemia, pre-eclampsia/eclampsia, and malnutrition, but the acute risk for adverse outcomes is much higher. Our study highlighted the complexity of assuring in a low-resource context that women who have this condition receive the necessary care.

The data highlighted several health systems and individual-level barriers to care. The high risk associated with malpresentation underlies the value of tertiary care, but the impracticality of the recommendation shone through the experiences of numerous women. Some facilities in this area abide by a blanket referral protocol for non-cephalic presentation as reported elsewhere [14] and hinted by interviewees, but the arduousness of completing that referral puts into question the clinical value. Both lower-level clinicians and families face the reality of the risk associated with the lower capacity available in greater proximity and the risk associated with the journey for tertiary care, without a guarantee of timely and high quality care.

This then puts into question how and whether to intervene on the lack of risk perception toward non-cephalic births among some women in the community. While recognizing that the high and acute risk of non-cephalic births could inspire women to seek higher-level care, neither the households nor frontline health workers have the ability to calculate the time, financial, and health cost-benefit of seeking tertiary care, with the care-seeking unknowns that exist in this context. This issue also applies to the value of diagnostics. Most women interviewed for the study were unaware of the condition prior to delivery, which was triangulated through quantitative data [14]. Ultrasonography is the only gold standard method of detecting non-cephalic presentation prior to delivery, and access and utilization are sparse in low-resource settings. In our study area, only about a quarter of women received an obstetric ultrasound exam during their most recent pregnancy [14]. While early diagnosis could translate to earlier care-seeking, the distance barrier and the possibility of poor care still remain and ultrasonography also has high associated cost. These points may be driving the mixed evidence behind ultrasonography as a maternal and neonatal intervention in low-income settings; the literature has reflected on the lack of population-level impact on health outcomes of ultrasonography, [15] but other studies have reported positive impact like change in clinical management [16].

Bhutta et al. reported that planned C-section for term breech presentation has supporting evidence of reducing stillbirths in low-resource settings, and also highlighted the potential for task shifting in areas where doctors are not readily available to perform the surgery [17]. Keeping in mind how critical and essential C-section capacity is for a proportion of all pregnancies, the risk-benefit of introducing a surgical intervention in low-resource settings should be seriously vetted.

In Nepal, Skilled Birth Attendants are trained in handling vaginal breech delivery, and are in theory available at every lower-level health facility. Especially for women who face immense barriers to tertiary care, these frontline workers could serve a critical role in fetal and maternal survival. One of our interviewees vividly described the poor clinical management that she attributed her child’s death to. Existing literature has also highlighted facility mismanagement related to non-cephalic presentation. A report by UNFPA and EngenderHealth described observations from Chad that if the traditional birth attendant during delivery cannot feel the head of the baby due to poor positioning, she will hold the woman by the ankles and shake her in the hopes that the baby will rotate, a behavior that could potentially lead to prolonged labor or obstetric fistula [18]. Several other studies have highlighted improper or inconsistent management of breech deliveries [19–21]. These issues flag both an issue of respectful care, but perhaps also the difficult task of training individuals and maintaining skills for less frequent, but dangerous, conditions like non-cephalic birth.

Related to mismanagement, another pervasive theme was the use of injections during labor and delivery. Injections appeared to be used to aid or quicken labor and delivery. Based on unpublished data from a previous study, we suspect that many of the injections were uterotonics (personal communication, Joanne Katz). Clinically, uterotonics can be used to help induce contractions and are used while monitoring the fetus; however, participants seemed to describe their use as indiscriminate – not a decision based on clinical need, but standard practice. A literature review has highlighted this issue, with use of uterotonics ranging widely from 1 to 69% during home births in low- and middle-income countries [22]. Such haphazard use of uterotonics could lead to negative health consequences, which has been previously observed in our study area [23]. Improper usage has been reported elsewhere as well [24, 25]. Other qualitative studies have highlighted how women associate uterotonics with positive effects on delivery, [22] a perspective shared by many of our interviewees. This requires careful consideration of how to differentiate positive clinical interventions from possible harmful ones.

A strength of our study is that the prospective data collection of the parent study allowed us to identify women who experienced this specific event at the time of delivery. The interviews were conducted within a few months of delivery, minimizing recall bias. Also, the organization through which these interviews were conducted has good rapport with the community, having worked there for over 25 years. A weakness of the study is that we did not capture health providers’ perspectives on non-cephalic presentation and their thought processes related to providing versus referring for care. Also, our interviewers were conducting qualitative research for the first time. The lack of experience likely impacted the interviewers’ ability to develop questions beyond the interview guide and probe without leading.

## Conclusions

Non-cephalic presentation is a predictor of adverse pregnancy outcomes, with high associated risk of fetal and neonatal death in low-resource settings. Our findings suggest the mixed quality and availability of care associated with this condition, and that there is no consistent or pervasive understanding of the risk of and care for non-cephalic births in rural Nepal. The high risk and recommended tertiary care associated with non-cephalic birth presents a major dilemma in low-resource settings; the logistical challenges and the mixed quality of care leave both households and frontline health workers with difficult decisions. While governments and public health stakeholders strive to improve the quality of and access to the formal health system, those players must also be sensitive to the context in promoting care-seeking.

## Additional files

Additional file 1: In-depth interview guide. This contains the in-depth interview guide in English and the local language of Maithili. (PDF 117 kb)

Additional file 2: Focus group discussion guide. This contains the focus group discussion guide in English and the local language of Maithili. (PDF 71 kb)

## Abbreviations

FGD: Focus group discussion
IDI: In-depth interview
