# Supplementary material for: Perceptions, careseeking, and experiences pertaining to non-cephalic births in rural Sarlahi District, Nepal: a qualitative study
Source: BMC Pregnancy Childbirth. 2018 Apr 10;18:89. doi: 10.1186/s12884-018-1724-2 (PMC5894138; doi:10.1186/s12884-018-1724-2)
Supplement: Supplementary file 1 — In-depth interview guide. This contains the in-depth interview guide in English and the local language of Maithili. (PDF 117 kb) [file 12884_2018_1724_MOESM1_ESM.pdf]

Supplementary Material 1: Interview guides

Interview Guide - WOMAN

We are interviewing women who had a baby who was delivered upside down, and we are trying to understand how to best care for mothers and babies who have these experiences. Thank you for agreeing to do the interview.

We will start the interview now. I am turning on the recorder.

हमनी सब यीअन्तरर्वाता उल्टा बच्चा पैदा भेल माई सबसँगे कर रहलछी और हमनी केनाके ऊ माईसब और बच्चासबके निमन देखभाल कर सकैछी कहके ओकर बारेमे बुझेके कोशिस कर रलहछी। आहाँके बहुत–बहुतधन्यवाद अन्तरर्वाता के लेल(राजी) सहमतहोएके लागि ।

अबहमनीअन्तरवार्ता सुरु करैछी और हम रेकर्डर खोल रहलछी ।

BACKGROUND

Let's start with telling me a little about yourself.
How old are you?
Who is in your family?
How many children do you have?
How old were you when you had your first child?

अब हम आहाँके बारेमे कुछ बातचित सुरु करैछी ।

1)आहाँ कैई बर्ष के होयली?

2)आहाँ के परिवारमे कोन- कोन हैई ?

3)आहाँके कईगो बेटा-बेटी हैई ?

4)पहिला लैकाहोएल बेरिमे आहाँ कैई बरिसके रहली?

PREGNANCY

Now, I want to ask you questions that are about your most recent pregnancy.

Can you tell me a little about your most recent pregnancy?

अब हम आहाँके हाल अन्तिम बेरिके गर्भवति समयके बारेमे किछो प्रश्नसब पुछेला चाहिछी

1) आहाँके हालसाल (अन्तिम पटक)के गर्भवतीअवस्थाके बारेमे किछो बतादुन

DURING LABOR AND DELIVERY

1) Can you tell me about what happened during labor and delivery?

---PRB---Can you tell me more about this / the problems that happened?

---PRB--- Can you tell me what you did to handle the problems that happened during labor and delivery?

---PRB---What roles did your family members play during labor and delivery?

2) If you know, what part of the baby came out first?

3) Can you tell me about if the baby coming upside down affected your delivery?

---PRB--- Can you tell me about the condition of your baby after delivery?

FOR ALIVE BABIES ---PRB---Can you tell me about the condition of the baby now?

FOR DEAD BABIES ---PRB---Can you tell me a little about what the reasons you think that your baby died / was born dead?

4) Where did you want to deliver the baby?

5) How did you decide to deliver at home / facility?

6) How did you decide not to deliver at home / facility?

1)लैकाहोएबालापिरा सुरु भेल बेरिसे लैका पैदा भेल समय भितरमे कथि -कथि भेलै ओकर बारेमे बतादेसकैछी ।

---PRB---उ समस्या सबके बारेमे आहाँहमरा आउरो कुछ बतादे सकैछी ?

---PRB---कोनो समस्या भेल हैत ऊ समस्या सब सुल्झाईके लेल कथि -कथि कैली उ सब बतादिउन।

---PRB---बच्चापैदाहोएबाला पिडा सुरु भेलबेरसे बच्चा पैदा नहोए बेरतकआहाँके परिवारके लोकसबके केहनभुमिका रहलै ?

2)अगर आहाँके मालुम है त आहाँके लैका जन्मीछै त लैका के शरिरके कोन भागअगारी निकल रहलै?

3)उल्टा लैकाजन्मेके कारणसे आहाँ केहन -केहन समस्यासबभोग्लीओकर बारेमे बतादेसकैछी ?

---PRB---लैकाजन्मे बेरीमे लैका के अवस्था कैसन रहलै ?यकर बारेमे कथियो बतादेसकैछी ?

FOR ALIVE BABIES ---PRB---लैकाके अखुन्तीके अवस्थाके बारेमे बता देसकैछी ?

FOR DEAD BABIES ---PRB---आहाँके बिचारमे कथि होएलकारणसे लैका मरल या मरल जन्मलहोतै?यकरा बारेमे हमरा तनकाबतासकैछी ?

4)**आहाँ काहाँ लैकाजन्माएला चाहैछी** ?

5) आहाँ लैका <u>**घरमे/अस्पतालमे**</u> जन्माए बाला निर्णय केना कैली या कथि कारण से कैली?

6) आहाँ लैका <u>**अस्पतालमे / घरमे**</u> जन्माए बाला निर्णय कथि कारण से नकैली ?

<u>**BEFORE LABOR AND DELIVERY**</u>

1) Did you know that your baby was upside down before delivery?

---PRB--- If yes, how did you find out?

---PRB---Did you do anything after you found out that the baby is upside down?

2) Can you tell me about any problems you experienced during the pregnancy?

---PRB--- How were the problems handled?

3) Is there anything else you would like to tell me about the time of pregnancy?

---PRB--- How about care she received from family, traditional healers, or health facility?

1)आहाँके लैका उल्टा है कहके उबाला बात लैकाजन्मेसे पहिले मालुम रहलै ?

---PRB---अगर मालुम रहलै त केनामालुम भेल रहलै ?

---PRB---लैका उल्टा है कहके मालुमहोएला के बादआहाँ कुछो कैले रहली?

2) आहाँगर्भवतीहोएल बेरीमे भोगलकोनो समस्या सबके बारेमे बतादेसकैछी?

---PRB---उ समस्यासब केनाकके सुल्भैली?

3) आउरो किछो हई गर्भवती समयके (बच्चा होई बाला पिडा सुरु होई से पहिले) बातसब जोन आँहा हमरा कहेला / बताबेला चाहिछी?

---PRB--- परिवारसे , धामी , भक्तासबसे या स्वास्थ्य संस्थासे केहन केहन सेवा सुबिधासब लेली या मिललै?

UPSIDE DOWN BABIES

1) Have you had any previous pregnancies when the baby was delivered upside down?

---PRB---If so, can you tell me a little about that experience?

---PRB---Did you know that your baby was upside down before delivery?

---PRB---If yes, how did you find out?

---PRB---Did you do anything after you found out that the baby is upside down?

---PRB---If you know, what part of the baby came out first?

---PRB--- Can you tell me about if the baby coming upside down affected your delivery?

2) Sometimes babies are born upside down. What did you know about babies that are born upside down before the delivery of this upside down baby?

---PRB--- What can be done if the child is upside down?

---PRB---What can be done during delivery for a child that is upside down?

3) Have you heard of any causes to babies being born upside down?

1) यैसे पहिले आहाँके कोनो लैका उल्टा जन्मल रहलै ?

---PRB---अगर रहलै त ओई समयके भोगल बातसबबतादेसकैछी ?

---PRB---आहाँके लैका उल्टा है कहके उबाला बात लैकाजन्मेसे पहिले मालुम रहलै ?

---PRB---अगर मालुम रहलै त केनामालुम भेल रहलै ?

---PRB---लैका उल्टा है कहके मालुमहोएला के बादआहाँ कुछो कैले रहली?

---PRB---अगर आहाँके मालुम है त आहाँके लैका जन्मलै त लैकाके शरिरके कोन भागअगारी निकल रहलै ?

---PRB--- उल्टा लैकाजन्मेके कारणसे आहाँ केहन -केहन समस्यासबभोग्लीओकर बारेमे बतादेसकैछी ?

2) कहियो -कहियो लैका उल्टा भि जन्मिछै, अई लैकाजन्मेसे पहिले आहाँके उल्टा जन्मल लैकाके बारेमे किछो जानकारी रहलै या सुन्ले रहली?

---PRB---अगर लैका उल्टा है त कथि करे सकिछै ?

---PRB---उल्टा लैकाके जन्मे बेरिमे कथि करे सकैछी?

3) आहाँ वईसन किछो कारण सबके बारेमे सुनले छी जोनसे लैका उल्टा होईछै?

CONCLUSION

Do you have anything else you would like to add to the interview before we finish?

Thank you so much for your time. I am now turning off the recorder and we are now finished with the interview.

1) आहाँके अन्तवार्ता ओराएसे पहिले दोसर कोनो बात कहके वा पुछेके मन हवे ?

हमनीके यतना समय देलीओइके लेल आहाँके बहुत-बहुत धन्यवाद ।

अब हमनी अन्तिममे छी और हम रेकर्डर बन्द करे जारहलछी ।

Interview guide – <u>FEMALE FAMILY MEMBER</u>

We are interviewing family members of women who had a baby who was delivered upside down, and we are trying to understand how to best care for mothers and babies who have these experiences. Thank you for agreeing to do the interview.

We will start the interview now. I am turning on the recorder.

हमनी सब यी अन्तरर्वाता उल्टा बच्चा पैदा भेल माई सब सँगे कर रहलछी और हमनी केनाके ऊ माईसब और बच्चा सबके निमन देखभाल कर सकैछी कहके ओकर बारेमे बुझेके कोशिस कर रहछी । आहाँके बहुत–बहुत धन्यवाद अन्तरर्वाता के लेल(राजी) सहमत होएके लागि ।

अब हमनी अन्तरवार्ता सुरु करैछी और हम रेकर्डर खोल रहलछी ।

BACKGROUND

Let's start with telling me a little about yourself.
How old are you?
Who is in your family?
How many children do you have?
How old were you when you had your first child?

अब हम आहाँके बारेमे कुछ बातचित सुरु करैछी होतै

1) आहाँ कैई बर्ष के होयली?

2) आहाँ के परिवारमे कोन- कोन हैई ?

3) आहाँके कईगो बेटा-बेटी हैई ?

4) पहिला लैकाहोएल बेरिमे आहाँ कैई बरिसके रहली?

PREGNANCY

Now, I want to ask you questions that are about your [daughter-in-law, daughter, sister-in-law, sister's] most recent pregnancy.

Can you tell me a little about her most recent pregnancy?

अब हम आहाँके........के हाल अन्तिम बेरिके गर्भवति समयके बारेमे कुछे प्रश्नसब पुछेला चाहिछी

1) आहाँके....... के हालसाल (अन्तिम पटक)के गर्भवती अवस्थाके बारेमे किछो बतादेसकैछी?

DURING LABOR AND DELIVERY

1) Can you tell me about what happened during labor and delivery?

---PRB---Can you tell me more about this / the problems that happened?

---PRB--- Can you tell me what you did to handle the problems that happened during labor and delivery?

---PRB---What roles did your family members play during labor and delivery?

2) If you know, what part of the baby came out first?

3) Can you tell me about if the baby coming upside down affected her delivery?

---PRB--- Can you tell me about the condition of the baby after delivery?

FOR ALIVE BABIES ---PRB---Can you tell me about the condition of the baby now?

FOR DEAD BABIES ---PRB---Can you tell me a little about what the reasons you think that the baby died / was born dead?

4) Where did you want your [daughter-in-law, daughter, sister-in-law, sister] to deliver the baby?

5) How did you decide to deliver at [home / facility]?

6) How did you decide not to deliver at [home / facility]?

1) आहाँके .... लैका होएबाला पिरा सुरु भेल बेरिसे लैका पैदा भेल समय भितरमे कथि -कथि भेलै ओकर बारेमे कुछो बतादेसकैछी?

---PRB--- उ समस्या सबके बारेमे आहाँ हमरा आउरो कुछ बतादेसकैछी ?

---PRB--- कोनो समस्या भेल हैत ऊ समस्या सब सुल्झाईके लेल कथि -कथि कैली उ सब बतादिउन ।

---PRB--- बच्चा पैदा होएबाला पिडा सुरु भेल बेरसे बच्चा पैदा नहोए बेरतक आहाँके परिवारके लोकसबके केहन भुमिका रहलै ?

2) अगर आहाँके मालुम है त आहाँके ......के लैका जन्मीछै त लैका के शरिरके कोन भागअगारी निकल रहलै ?

3) उल्टा लैकाजन्मेके कारणसे आहाँके...... केहन-केहन समस्या सबके भोग्ले रहलै ओकर बारेमे बतादेसकैछी ?

---PRB--- लैकाजन्मे बेरीमे लैकाके अवस्था कैसन रहलै ?यकर बारेमे कथियो बतादेसकैछी ?

FOR ALIVE BABIES ---PRB--- लैकाके अखुन्तीके अवस्थाके बारेमे बतादेसकैछी ?

FOR DEAD BABIES ---PRB---आहाँके बिचारमे कथि होएलकारणसे लैका मरल या मरल जन्मलहोतै ?यकरा बारेमे हमरा तनका बतासकैछी?

4) आहाँ अपन………… के लैका कहमा हो कहके चाहित रहली?

5) आहाँके …….के लैका <u>घरमे/अस्पतालमे</u> जन्माए बाला निर्णय केना कैली या कथि कारण से कैली?

5) आहाँके …….के लैका <u>अस्पतालमे / घरमे</u> जन्माए बाला निर्णय कथि कारण से नकैली ?

<u>BEFORE LABOR AND DELIVERY</u>

1) Did you know that her baby was upside down before delivery?

   ---PRB--- If yes, how did you find out?

   ---PRB---Did you do anything after you found out that the baby is upside down?

2) Can you tell me about any problems she experienced during the pregnancy?

   ---PRB--- How were the problems handled?

3) Is there anything else you would like to tell me about the time of pregnancy?

   ---PRB--- How about care she received from family, traditional healers, or health facility?

1) आहाँके …….के लैका उल्टा है कहके उबाला बात लैकाजन्मेसे पहिले मालुम रहलै ?

   ---PRB---अगर मालुम रहलै त केना मालुम भेल रहलै ?

   ---PRB--- लैका उल्टा है कहके मालुम होएला के बाद आहाँ कुछो कैले रहली?

2) ऊ गर्भवती होएल बेरीमे भोगल कोनो समस्यासबके बारेमे बतादेसकैछी?

   ---PRB---उ समस्यासब केना कके सुल्भैली?

3) आउरो किछो हई आहाँके …….के गर्भवती समयके (बच्चा होई बाला पिडा सुरु होई से पहिले) बातसब जोन आँहा हमरा कहेला / बताबेला चाहिछी?

---PRB--- परिवारसे , धामी , भक्तासबसे या स्वास्थ्य संस्थासे केहन केहन सेवा सुबिधासब लेली या मिललै?

UPSIDE DOWN BABIES

1) Has she had any previous pregnancies when the baby was delivered upside down?

---PRB---If so, can you tell me a little about that experience?

---PRB---Did you know that her baby was upside down before delivery?

---PRB---If yes, how did you find out?

---PRB---Did you do anything after you found out that the baby is upside down?

---PRB---If you know, what part of the baby came out first?

---PRB--- Can you tell me about if the baby coming upside down affected her delivery?

2) Sometimes babies are born upside down. What did you know about babies that are born upside down before the delivery of this upside down baby?

---PRB--- What can be done if the child is upside down?

---PRB---What can be done during delivery for a child that is upside down?

3) Have you heard of any causes to babies being born upside down?

1) यैसे पहिले आहाँके ....के कोनो लैका उल्टा जन्मल रहलै ?

---PRB--- अगर रहलै त ओई समयके भोगल बातसब बतादेसकैछी ?

---PRB--- आहाँके .....के लैका उल्टा है कहके उबाला बात लैका जन्मेसे पहिले मालुम रहलै?

---PRB--- अगर मालुम रहलै त केना मालुम भेल रहलै ?

---PRB--- लैका उल्टा है कहके मालुम होएलाके बाद आहाँ कुछो कैले रहली?

---PRB--- अगर आहाँके मालुम है त आहाँके ....के लैका जन्मलै त लैकाके शरिरके कोनभाग अगारी निकल रहलै ?

---PRB--- उल्टा लैका जन्मेके कारणसे आहाँके...... केहन-केहन समस्यासब भोग्ले रहलै ओकर बारेमे बतादेसकैछी ?

2) कहियो-कहियो लैका उल्टा भि जन्मिछै,अई लैकाजन्मेसे पहिले आहाँके उल्टा जन्मल लैकाके बारेमे किछो जानकारी रहलै या सुन्ले रहली?

---PRB--- अगर लैका उल्टा है त कथि करे सकिछै ?

---PRB--- उल्टा लैकाके जन्मे बेरिमे कथि करे सकैछी?

3) आहाँ वईसन किछो कारण सबके बारेमे सुनले छी जोनसे लैका उल्टा होईछै?

CONCLUSION

Do you have anything else you would like to add to the interview before we finish?

Thank you so much for your time. I am now turning off the recorder and we are now finished with the interview.

1) आहाँके अन्तवार्ता ओराएसे पहिले दोसर कोनो बात कहके वा पुछेके मन हवे ?

हमनीके यतना समय देली ओइके लेल आहाँके बहुत-बहुत धन्यवाद ।

अब हमनी अन्तिममे छी और हम रेकर्डर बन्द करे जारहलछी।
