# Supplementary material for: Perceptions, careseeking, and experiences pertaining to non-cephalic births in rural Sarlahi District, Nepal: a qualitative study
Source: BMC Pregnancy Childbirth. 2018 Apr 10;18:89. doi: 10.1186/s12884-018-1724-2 (PMC5894138; doi:10.1186/s12884-018-1724-2)
Supplement: Supplementary file 2 — Focus group discussion guide. This contains the focus group discussion guide in English and the local language of Maithili. (PDF 71 kb) [file 12884_2018_1724_MOESM2_ESM.pdf]

Supplemental Material 2:
Focus group guide

General pregnancy care

Today we are going to ask you questions about problems women in your community experience during pregnancy and how they are handled.

आज हामी तपाईको समुदायमा रहनुभएको गर्भवती महिलाले कस्ता-कस्ता समस्या भोग्नु भयो अनि ती समस्याहरु लाई कसरी समाधान गर्नुभयो त्यसको बारेमा छलफल गर्छौं हुन्छ ।

What are some things that are done for a pregnant woman to make sure she and the baby are healthy during pregnancy?

गर्भावस्थामा गर्भवती आमा र बच्चा दुवै स्वस्थ छन् भन्ने कुरा थाहा निश्चित गर्न तपाईहरुको समुदायमा के -के गरिन्छ ?

What are some things during pregnancy that suggest that the pregnant woman or the baby may have health problems?

गर्भवती आमा र बच्चाको स्वास्थ्यमा समस्या छ होला भन्ने कुरा कसरी थाहा हुन्छ ?

---PRB--- How about during labor and delivery?

सुत्केरी बेथा लागेदेखी बच्चा नहुन्जेल सम्म ?

---PRB--- How about before labor and delivery?

सुत्केरी बेथा लाग्नु भन्दा अगाडीको अवस्था ?

How does one deal with those issues?

त्यस्ता समस्याहरुलाई कसरी समाधान गर्नुहुन्छ?

Fetal presentation

Sometimes babies are born upside down. We are now going to ask you some questions about that.
कहिले काँही बच्चा उल्टो पनि जन्मिन्छन् ? आज हामी यसैको बारेमा केही कुराहरु गर्न जाँदैछौं ।

What do you know about babies that are born upside down?
उल्टो जन्मिएको बच्चाको बारेमा तपाईहरुलाई के थाहा छ त्यसको बारेमा बताईदिन सक्नुहुन्छ ?

How can one tell prior to delivery that a baby is coming out upside down?
गर्भावस्थामा बच्चा उल्टो छ भन्ने कुरा कसरी थाहा पाउन सकिन्छ ?

What can be done if the child is upside down?
गर्भावस्थामा यदि बच्चा उल्टो छ भने के गर्न सकिन्छ ?

What can be done during delivery for a child that is upside down?

उल्टो बच्चा जन्मिने बेलामा के गर्न सकिन्छ ?

Do you know any friends or neighbors who had a baby born upside down?
तपाईहरुले छिमेकी वा आफ्ना आफन्तहरुको उल्टो बच्चा जन्मिएको सुन्नु भएको वा थाहा पाउनु भएको छ ?

---PRB--- What was their experience like?
उहाँहरुको अनुभव कस्तो थियो ?

What causes babies to come out upside down?
के तपाईहरुलाई थाहा छ के कारणले बच्चा उल्टो जन्मिएको होला ?

Do you have anything else to add about upside down babies?
अब हामी अर्को बिषयमा कुराकानी गर्न जाँदैछौँ तपाईहरुलाई उल्टो जन्मिने बच्चाहरुको बारेमा केही कुरा भन्नु छ ?

Delivery preferences

Some people in your community choose to deliver a baby at home. Some people choose to deliver a baby at a facility.
तपाईँको समुदायमा केही महिलाहरु घरमा बच्चा जन्माउन चाहनुहुन्छ , केही महिला अस्पतालमा बच्चा जन्माउन चाहनुहुन्छ ,अव हामी यसै विषयमा केही छलफल गरौँ ।

What are some reasons women prefer home deliveries?
के कति कारणले गर्दा महिलाहरु घरमा बच्चा जन्माउन रुचाउनुहुन्छ ?

What are some reasons women prefer facility deliveries?
के कति कारणले गर्दा महिलाहरु अस्पतालमा बच्चा जन्माउन रुचाउनु हुन्छ :?

What do people in your community think about cesarean section operations?
तपाईहरुको समुदायमा ठूलो अपरेशन गरेर बच्चा पाउनु (जन्माउनु) लाई कस्तो रुपमा हेरिन्छ वा कस्तो रुपमा लिईन्छ ?

Conclusion

Is there anything else you would like to say before we finish?

हामी आजको छलफलको अन्तमा छौँ । यदि तपाईहरुलाई यसको बिषयमा भन्न मन लागेको अझै अरु केही छ भने भन्न सक्नु हुन्छ ?

Thank you so much for your time today.
